# Reproducibility of patient setup by surface image registration system in conformal radiotherapy of prostate cancer

**DOI:** 10.1186/1748-717X-4-9

**Published:** 2009-02-22

**Authors:** Marco Krengli, Simone Gaiano, Eleonora Mones, Andrea Ballarè, Debora Beldì, Cesare Bolchini, Gianfranco Loi

**Affiliations:** 1 Department of Radiotherapy, University Hospital Maggiore della Carità, Novara, Italy; 2 Department of Clinical and Experimental Medicine and Biotechnology Centre for Applied Medical Research, University of Piemonte Orientale, Novara, Italy; 3 Department of Medical Physics, University Hospital Maggiore della Carità, Novara, Italy

## Abstract

**Background:**

The reproducibility of patient setup for radiotherapy is based on various methods including external markers, X-rays with planar or computerized image acquisition, and, more recently, surface matching imaging. We analyzed the setup reproducibility of 16 patients affected by prostate cancer who underwent conformal radiotherapy with curative intent by using a surface image registration system.

**Methods:**

We analyzed the setup reproducibility of 16 patients affected by prostate cancer candidates for conformal radiotherapy by using a surface image registration system. At the initial setup, EPID images were compared with DRRs and a reference 3D surface image was obtained by the AlignRT system (Vision RT, London, UK). Surface images were acquired prior to every subsequent setup procedure. EPID acquisition was repeated when errors > 5 mm were reported.

**Results:**

The mean random and systematic errors were 1.2 ± 2.3 mm and 0.3 ± 3.0 mm along the X axis, 0.0 ± 1.4 mm and 0.5 ± 2.0 mm along the Y axis, and 2.0 ± 1.8 mm and -0.7 ± 2.4 mm along the Z axis respectively. The positioning error detected by AlignRT along the 3 axes X, Y, and Z exceeded the value of 5 mm in 14.1%, 2.0%, and 5.1% measurements and the value of 3 mm in 36.9%, 13.6% and 27.8% measurements, respectively. Correlation factors calculated by linear regression between the errors measured by AlignRT and EPID ranged from 0.77 to 0.92 with a mean of 0.85 and SD of 0.13. The setup measurements by surface imaging are highly reproducible and correlate with the setup errors detected by EPID.

**Conclusion:**

Surface image registration system appears to be a simple, fast, non-invasive, and reproducible method to analyze the set-up alignment in 3DCRT of prostate cancer patients.

## Background

Accurate and repeatable patient setup is a pre-requisite for radiotherapy in order to limit the margin around the clinical target volume (CTV), i.e. the planning target volume (PTV), and consequently minimize the irradiation of healthy tissues responsible for early and late side effects. The reproducibility of external patient alignment is independent from the internal organ motion that can affect the position of the tumor with respect to the surrounding healthy tissues. Both aspects have to be taken into account as prescribed by the ICRU 62 document (ICRU62) that defined in this regard the setup margin and the internal margin around the CTV to obtain the final PTV.

For prostate cancer, as well as for other tumors, the optimization of the setup procedure as well as the definition of the internal organ motion has become of greater relevance over the last decade in relation to the implementation of highly conformal radiation techniques such as 3-dimensional conformal radiation therapy (3DCRT), intensity modulated radiation therapy (IMRT), and charged particle therapy [1,2].

The verification of patient setup can be performed by a number of methods of varying sophistication, including: using external markers; X-rays with planar or computerized image acquisition; and, more recently, surface matching imaging [3-6]. Of these methods, X-rays with planar (after implant of radio-opaque seeds) and computerized image acquisition are able to verify both patient setup and prostate position whereas surface matching imaging is directed to verify the patient setup only. The latter method has the potential advantage of being non invasive since no ionizing radiation is used. Moreover, a number of studies showed that the implementation of such technique allowed to obtain a high degree of precision for patient setup for breast and thoracic tumor locations [7-10].

In the present article, we analyzed the setup reproducibility of 16 patients affected by prostate cancer who underwent conformal radiotherapy with curative intent by using a surface image registration system.

## Methods

The system for surface image registration installed in the Department of Radiotherapy at the University Hospital "Maggiore della Carità" in Novara, Italy is presented and the acceptance tests preliminary to clinical activity are described. Then the procedure for image acquisition in a clinical series of 16 cases of prostate radiation treatment is reported and the methods of data analysis are described.

### Image acquisition system

**Figure 1:**
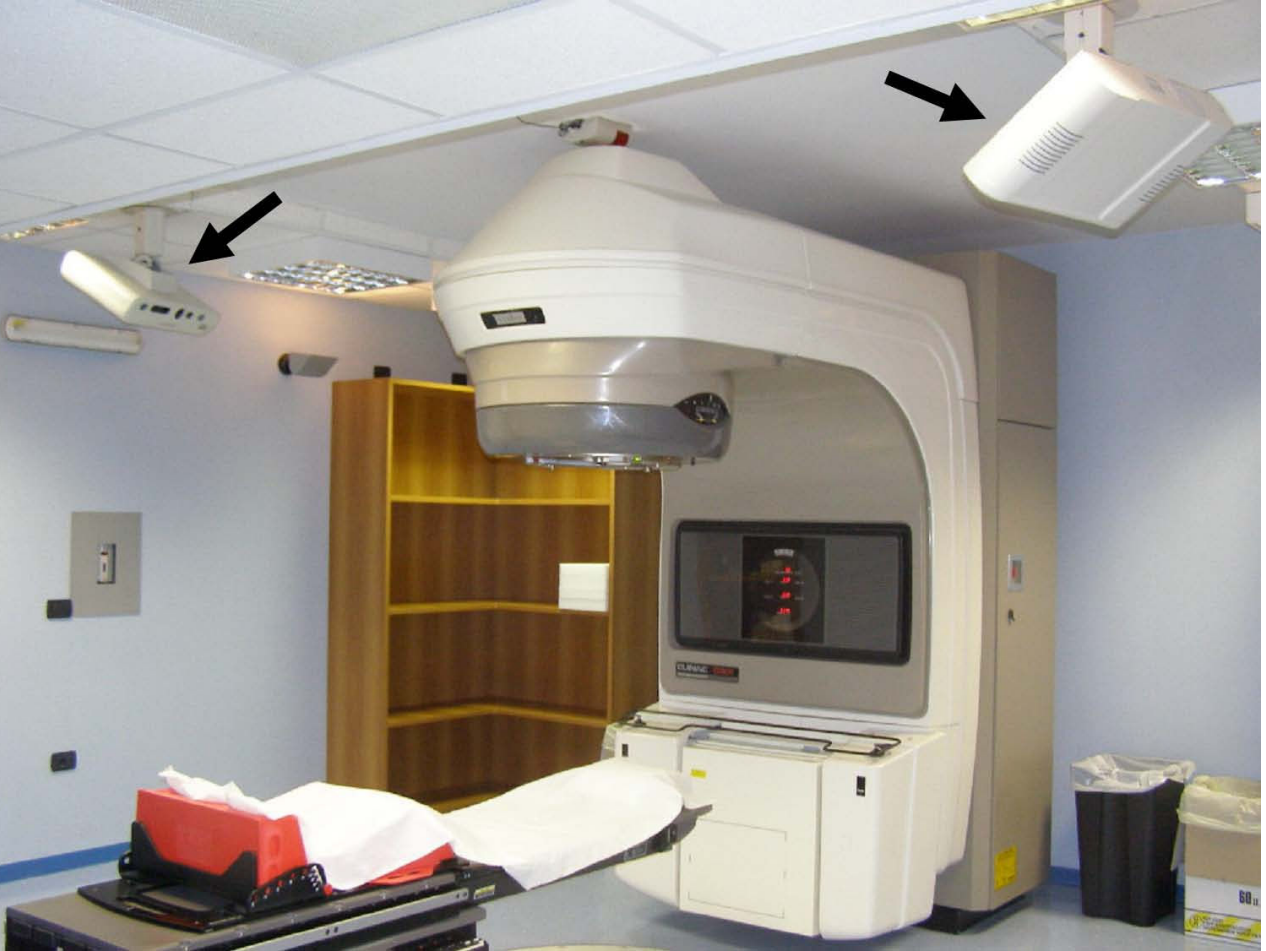
**Photograph of the two camera pods (black arrows) of the surface registration system, mounted on the ceiling of the treatment room**. The linear accelerator is also shown.

The commercially available 3D surface image registration system AlignRT (Vision RT, London, UK) was installed in a treatment room equipped with a linear accelerator with multileaf collimator and amorphous silicon electronic portal imaging device (EPID) (Figure 1). The AlignRT system consists of two imaging pods mounted on the ceiling under an oblique angle of 30° with respect to the treatment table [11,7]. Each pod containing two stereo-vision cameras, a texture camera, a clear flash, a flash used for speckle projection, and a slide projector for speckle projection, acquires 3D surface data over approximately 120° in the axial plane, from midline to posterior flank. The data are merged to form a single 3D surface image of the patient. The system includes software designed to facilitate patient setup by surface-model acquisition and alignment by surface matching with a reference. The reference image can be obtained at the time of first treatment session, in the simulator room equipped by a second imaging system, or by extraction of the surface image from CT data. In order to optimize the alignment process, the software is able to calculate the optimal rigid-body transformation (couch translation and rotation) that brings the surface model of the daily treatment fraction into congruence with the reference surface.

Before starting the clinical activity, a test was performed in order to verify the performance of the system in terms of precision and reproducibility of the measures. An anthropomorphic phantom was positioned on the treatment table and aligned with the three laser system of the treatment room. The known shifts of the treatment table along the three axes was checked by the AlignRT with measurements for each axis X, Y, and Z. The system demonstrated high accuracy and reproducibility with measured errors of less than 1 mm. A quality assurance procedure was adopted for the AlignRT system by daily checks to calibrate the cameras to the coordinates of the linear accelerator using a dedicated calibration plate with a printed grid.

### Clinical series

Sixteen patients aged from 61 to 78 years (median 73 years) were enrolled in the present study after obtaining informed consent following the rules of our institution. All patients were affected by prostate cancer with Gleason score ranging from 6 to 9 (median 7.5) and PSA level at diagnosis ranging from 2 to 75 ng/ml (median 12 ng/ml). The whole cohort had a body mass index (BMI) ranging from 19.5 to 29.1 (mean 23.7) and 4 patients with a BMI > 25 and were defined as overweight, following the definition adopted by the World Health Organization. Treatment consisted of 3D-conformal radiotherapy to a total dose of 70 – 76 Gy in 35 – 38 fractions of 2 Gy by a 6 coplanar conformal field technique over a period of 7 – 7 and a half weeks. The planning target volume (PTV) was obtained by a 10 mm expansion around the CTV except for the posterior margin where a 7 mm expansion was used towards the rectal wall. All patients were treated in supine position with partially filled bladder and empty rectum using a knee-ankle fixation device to facilitate setup reproducibility. Three skin tattoos, two lateral and one anterior, were marked for position verification by alignment to the 3 laser system. Simulation was performed by conventional simulator (Ximatron, Varian, Palo Alto, CA, USA) and spiral computed tomography (CT)-scan (Lightspeed, General Electric, Milwaukee, WI, USA) obtaining 5 mm slice thickness images spaced from L4 to 2 cm below the ischeal tuberosities. The images with DICOM 3 format were transferred to the treatment planning system (TPS) Pinnacle (Philips, Eindhoven, The Netherlands) by local network.

### Image acquisition

**Figure 2:**
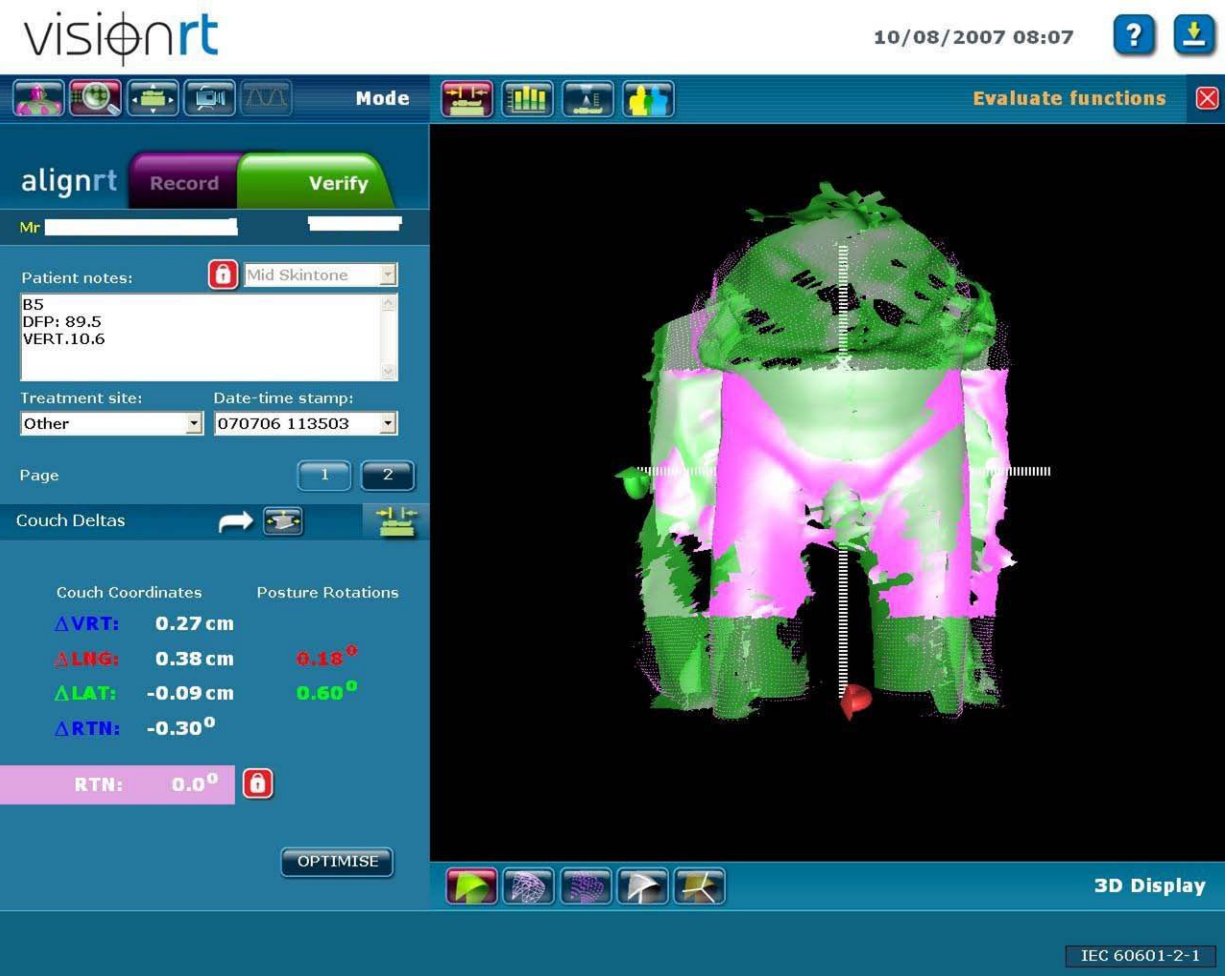
**Alignment of a daily image to the reference surface image**.

During the first treatment session, the patient was aligned by the laser system with the three skin tattoos and two orthogonal EPID images were acquired, typically anterior-posterior and latero-lateral. The images were matched with the digitally reconstructed radiographs (DRRs) from the CT simulation using a dedicated software (Vision, Varian, Palo Alto, CA, USA). The alignment was validated by a radiation oncologist on the basis of bone anatomy. At the same time, a reference surface image of the region of interest (ROI) of the patient was obtained and recorded by the AlignRT system. The ROI was defined as the lower abdomen from the umbilical region to the mid thighs. The image was aligned to the reference image using the surface information contained within the ROI. An example of surface alignment is shown in Figure 2.

Surface images were acquired during every setup procedure. When the error detected by the AlignRT system was > 5 mm, an EPID acquisition was obtained in order to verify the setup error along the three main axes (X: left-right, Y: anterior-posterior, Z: cranial-caudal). The tolerance threshold of 5 mm was chosen as it corresponded approximately to 2 standard deviations (SD) of the setup errors for prostate treatment detected in clinical practice at our institution by a previous study conducted comparing serial EPIDs with DRRs (SD on X axis = 2.2 mm, SD on Y axis = 1.7 mm, SD on Z axis = 2.6 mm) [12]. The errors along the 3 main axes calculated by means of Align RT were compared with the setup errors detected by the EPID images.

Rotational errors detected by the surface imaging system were not specifically analyzed in the present study and were neglected when < 1°.

### Statistical analysis

Systematic and random errors were calculated using the van Herk's formula [13] and reported as mean and standard deviation (SD). The percentage of error correction was calculated with two threshold levels: > 3 mm and > 5 mm. The latter threshold level > 5 mm was adopted in clinical practice as action level. The correlation between the positioning errors measured by AlignRT and those determined by EPID was performed by linear regression method.

## Results

**Figure 3:**
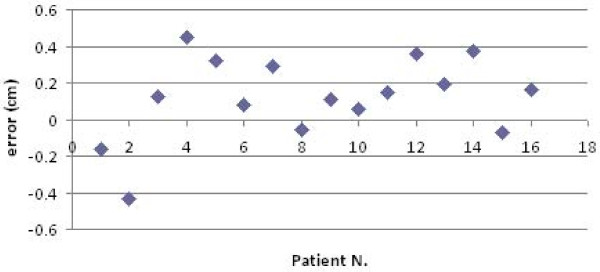
**Systematic errors detected by AlignRT along the X axis in the 16 patients**.

**Figure 4:**
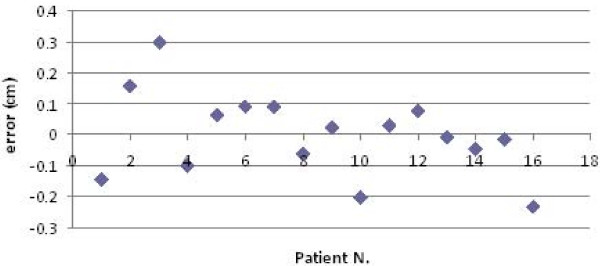
**Systematic errors detected by AlignRT along the Y axis in the 16 patients**.

**Figure 5:**
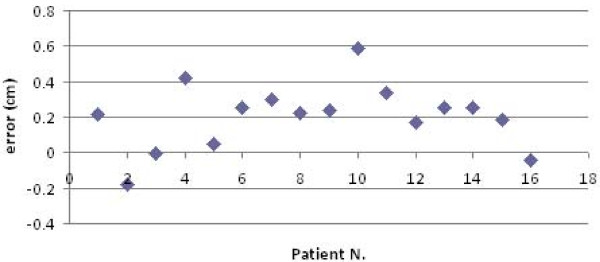
**Systematic errors detected by AlignRT along the Z axis in the 16 patients**.

**Figure 6:**
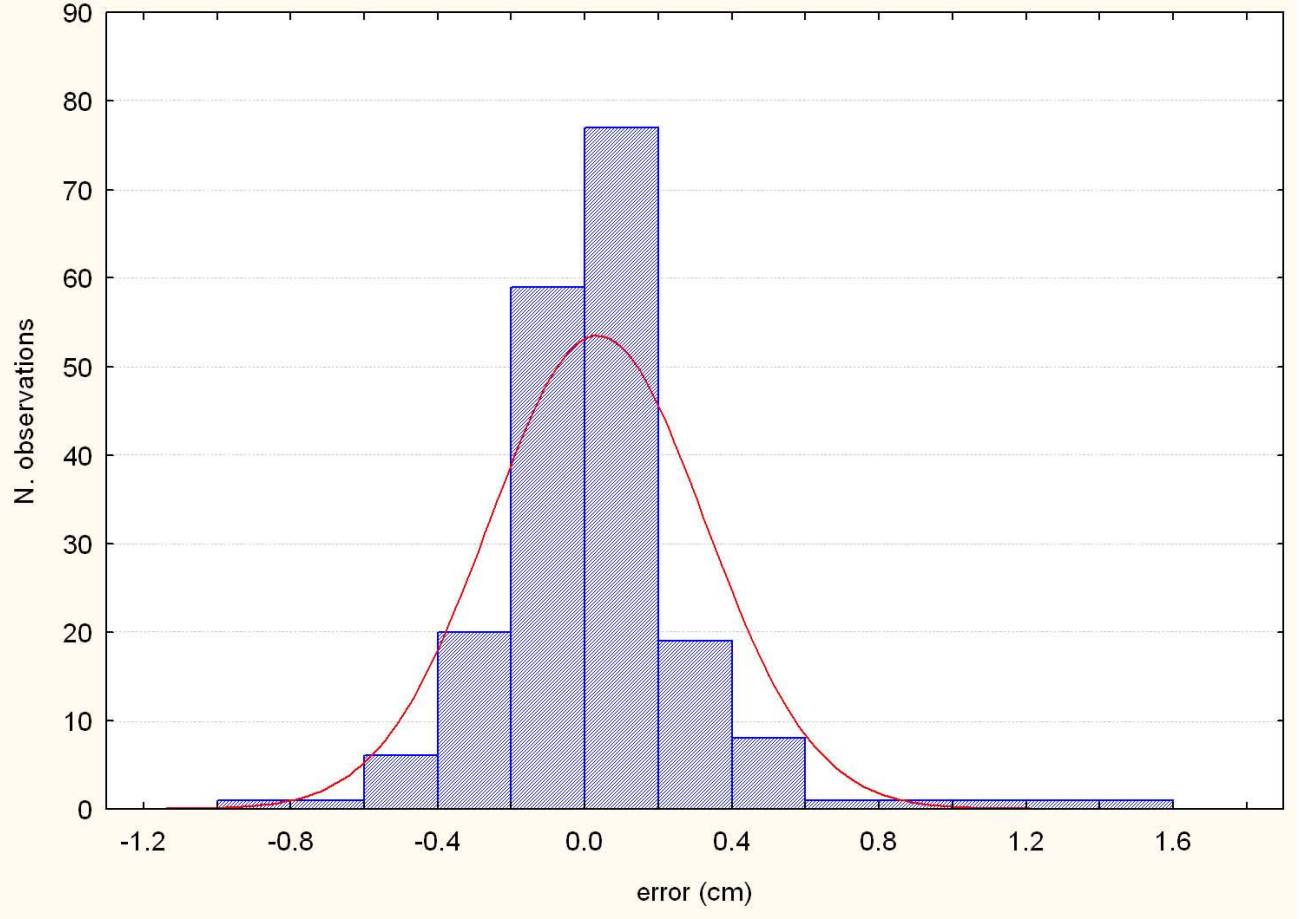
**Representation of the random errors detected by AlignRT along the X axis in the 16 patients**.

**Figure 7:**
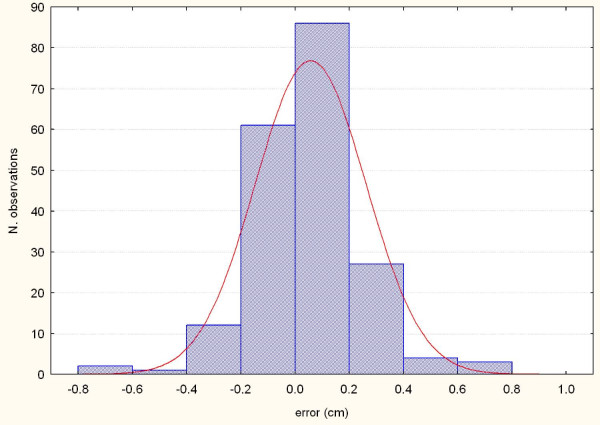
**Representation of the random errors detected by AlignRT along the Y axis in the 16 patients**.

**Figure 8:**
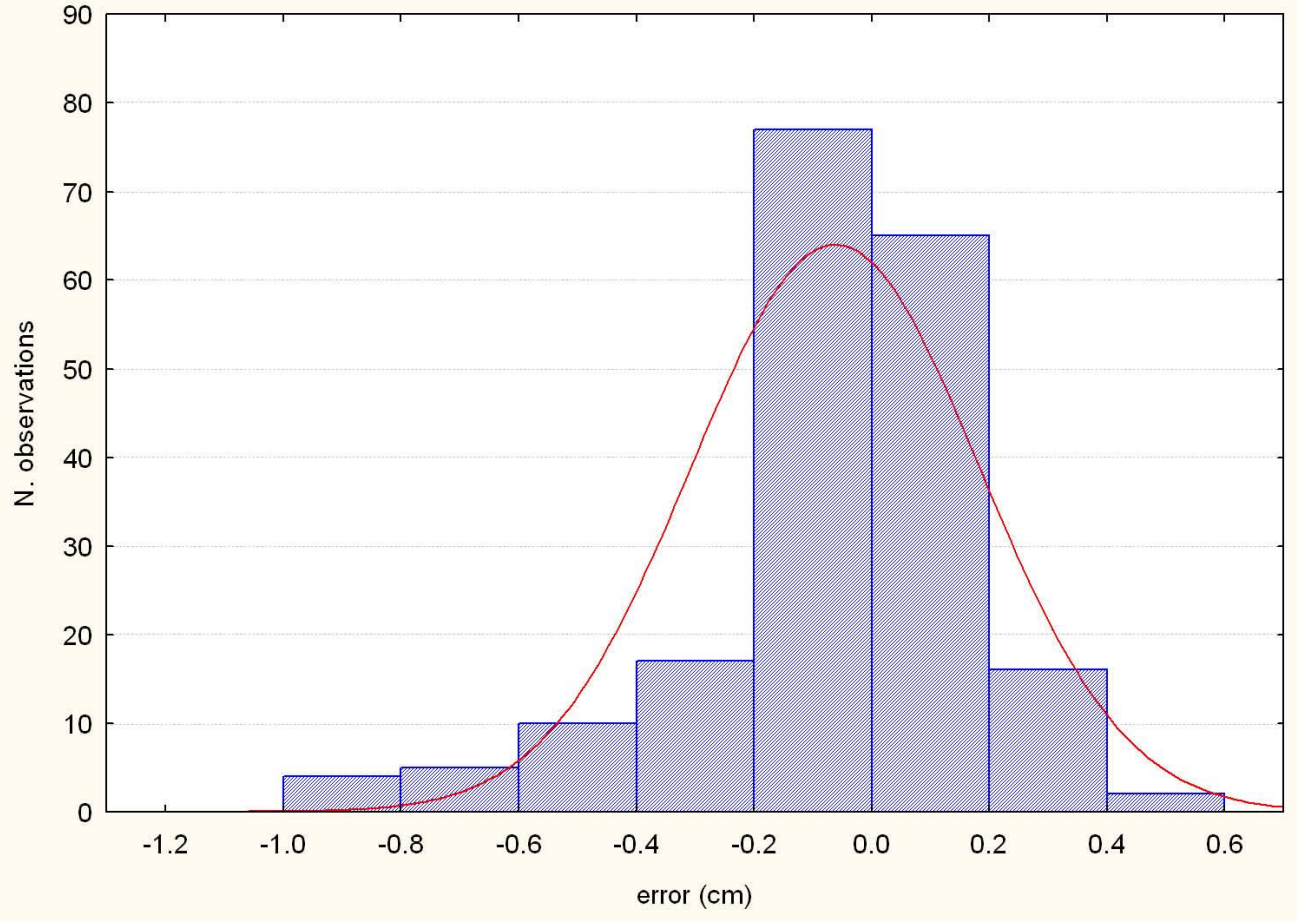
**Representation of the random errors detected by AlignRT along the X axis in the 16 patients**.

**Figure 9:**
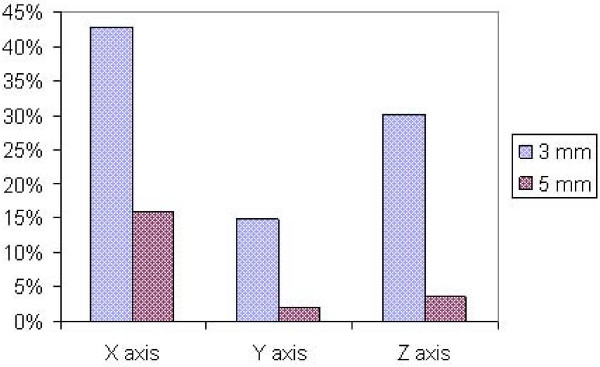
**Positioning errors detected by AlignRT along the 3 axes X, Y, and Z exceeding the values of 3 and 5 mm**.

The procedure for image acquisition and comparison with the reference image took about 30 seconds. The mean systematic and random errors detected by AlignRT along the three main axes are reported in Table 1 and in Figure 3, 4, 5, 6, 7 and 8. The positioning error detected by AlignRT along the 3 axes X, Y, and Z exceeded the value of 5 mm in 14.1%, 2.0%, and 5.1% of measurements and the value of 3 mm in 36.9%, 13.6%, and 27.8% of measurements respectively (Figure 9). Considering all the 16 patients, the positioning error exceeded at least once 5 mm in 11, 4, and 2 cases and the value of 3 mm in 14, 10, and 15 cases on the X, Y, and Z axis respectively. For the 4 overweight patients, the positioning error exceeded at least once 5 mm in 3/4 cases on the X axis and in 2/4 cases on the Y and Z axes.

**Table 1:** Systematic and random errors (mm) along the three main axes (X, Y, and Z) detected by AlignRT

**Axis**	**Systematic error (mean ± SD)**	**Random error (mean ± SD)**
X	1.2 ± 2.3	0.3 ± 2.9

Y	0.0 ± 1.4	0.5 ± 2.0

Z	2.0 ± 1.8	-0.7 ± 2.4

The correlation factor calculated by means of linear regression between the positioning errors measured by AlignRT and EPID images ranged from 0.77 to 0.92 with a mean of 0.85 ± 0.13.

## Discussion

A number of studies using video-surface imaging for patient setup verification have been published over the last few years [14,3,11,7,5,10]. Most of them were performed on breast cancer and intra-thoracic tumors and showed that surface imaging is a reliable method for patient position verification and may improve the precision of setup for breast cancer patients and reduce the effects of respiratory motion [7,9,10]. In the present study, we applied a surface image registration system to verify the position of prostate cancer patients during radiotherapy. This technique aims to verify the patient position before each treatment session and not the prostate position inside the body that may change in relation with rectum and bladder filling. In this sense, a correct patient setup can reduce the so-called setup margin but not the internal margin as defined by the ICRU 62 document (ICRU 62).

To our knowledge, only one study investigating the use of surface imaging for analysis of the setup of patients affected by pelvic malignancies and in particular by prostate cancer has been reported in the literature thus far [3]. In this study, Ploeger et al. (2003) [3] analyzed the left-right translation error in 22 prostate patients by both portal vision and video surface imaging. They reported that the largest contribution to the measured set-up errors was due to the set-up error of the bony anatomy while the SD of movement of the skin with respect to bony anatomy was estimated to be 1.1 mm. Furthermore, they observed that the correlation between the video setup error and the portal setup error was higher than the correlation between the marker position and the portal setup error and that the use of video recorded images may be able to reduce the number of setup corrections.

The present study aimed at analyzing the reproducibility of patients' setup by using the video-surface imaging registration system AlignRT in a series of 16 patients who were affected by prostate carcinoma and were candidates for curative conformal radiotherapy. In this study, we used the acquisition at the time of the first session as a reference image since we had not a surface image system in the simulation room and we decided to avoid any possible error in the matching of the CT reconstruction data and the Align-RT data. The latter procedure could actually be critical in relation to a change of coordinate system from CT to surface imaging system as already observed by Bert et al. [7] (Bert 2006). The images obtained by the AlignRT system correlated well with EPID images as shown by the linear regression between the positioning errors measured by AlignRT and EPID images that ranged from 0.77 to 0.92 with a mean of 0.85 and SD of 0.13. The analysis of systematic and random errors showed that the mean largest systematic error was found on the Z axis and the highest SD was found in the X axis, similarly to what observed by Kupelian et al. [1] in a recent series of 74 cases treated with helical tomotherapy. Also other authors found that errors along the X axis may have a SD higher than that along the other directions [15,3]. In particular, Ploeger et al. reported SD values of the systematic and random components of the set-up errors derived from the portal images in the left-right direction (X axis in our study) of 1.5 and 2.1 mm, respectively. Interestingly, they observed that when the set-up of the patients was retrospectively adjusted based on the video images, the SD of the systematic and random errors decreased to 1.1 and 1.3 mm, respectively. These values are slightly lower compared to the SD observed in the present series and to the values reported by Kupelian et al. [1] (Table 1). This difference may be related to the patient selection in terms of percentage of overweight cases and compliance to the procedure.

The distribution of positioning errors along the three main axes detected by the surface imaging system during all the treatment time span shows that the most frequent deviations from the threshold levels happened on the X axis (14%). Along the other axes, Y and Z, the errors greater than 5 mm, i.e. the action level used in clinical practice, occurred only in 2% and in 5% of cases respectively. This behavior may be related to the variations of patient profile mainly due to change in content of bowel and small intestine. As a matter of fact, we observed variations > 5 mm in the X axis in 3 out of 4 overweight patients. In this regard, Wong et al. [6] found a significantly larger shift in the lateral direction for the obese group in a series of 329 patients affected by prostate cancer. Although we did not specifically investigated this aspect because of the limited number of patients of our series, EPID might result more reliable than surface imaging in setup verification in overweight and obese patients.

As expected, the two different threshold values of 3 and 5 mm, considered in the present analysis (the threshold of 5 mm was adopted in clinical practice to correct the patient position), would have led to a different percentage of patient repositioning with a substantially higher correction rate for the action level of 3 mm (37%, 14%, and 28% for X, Y, and Z axis respectively) than for the action level of 5 mm (14%, 2%, and 5% for X, Y, and Z axis respectively). Based on these findings, the routinely daily use of surface imaging may lead to a reduction of the setup margin around the CTV, i.e. the PTV.

The results observed in this study in terms of setup error are substantially consistent with those reported by other authors using different setup control tools [4,1,6]. Kupelian et al. [1] in their series of 74 patients treated by helical tomotherapy found that setup errors > 5 mm occurred in 24% of fractions and this frequency increased to about 40% when setup errors > 3 mm were scored.

Since our study did not correlate surface images with CT images but only with EPID, we were not able to correlate our findings with the prostate itself but only with bony landmarks. Furthermore, we did not analyze other parameters that may affect the patient setup like rotation, intra-fraction motion, and breathing movements. These potential sources of errors could be studied by surface imaging system as suggested by Brahme et al. [5]. Rotational errors were not specifically analyzed in the present series but could be considered in a further study. Another interesting analysis could be performed by considering also non-rigid effects instead of analyzing only the shifts along the three main axes as a rigid relationship.

## Conclusion

In conclusion, the data from our study show that the setup measurements by surface imaging are reproducible and are in accord with the setup errors detected by EPID. Although further studies on larger patients' cohorts are needed to validate such an approach, surface image registration system appears to be a simple, fast, non-invasive, and promising method to analyze the set-up alignment of the patient, that can be used to define and whenever possible minimize the setup margin, in 3DCRT for prostate cancer.

## Abreviations

3DCRT: 3 dimensional conformal radiation therapy; CT: computed tomography; CTV: clinical target volume; DICOM: digital imaging and communications in medicine; DRR: digital reconstructed radiograph; EPID: electronic portal imaging device; Gy: Gray; IMRT: intensity modulated radiation therapy; MD: medical doctor; MRT: medical radiation technologist; PhD: physical doctor; PTV: planning target volume; ROI: region of interest; SD: standard deviation; TPS: treatment planning system; CI: confidence interval

## Competing interests

The authors declare that they have no competing interests.

## Authors' contributions

MK was the study coordinator, participated in the development of the study and drafted the manuscript. SM and CB were involved in data collection. GL and EM worked on analysis of data. AB and DB participated in the design of the study and contributed to write the manuscript. All authors read and approved the final manuscript.
